# Editorial: Colyn Crane-Robinson (1935–2023)

**DOI:** 10.1093/nar/gkad625

**Published:** 2023-07-26

**Authors:** Andrew J Bannister, Robert Schneider, Patrick Varga-Weisz

**Affiliations:** Gurdon Institute and Department of Pathology, University of Cambridge, Cambridge CB2 1QN, UK; Institute of Functional Epigenetics, Helmholtz Center Munich, Munich, Germany; Faculty of Biology, Ludwig Maximilian University (LMU) of Munich, Munich, Germany; School of Life Sciences, University of Essex, Colchester, CO4 3SQ, UK; International Laboratory for Microbiome Host Epigenetics, Department of Genetics, Evolution, Microbiology, and Immunology, Institute of Biology, University of Campinas, Campinas, Brazil

**Figure 1.**
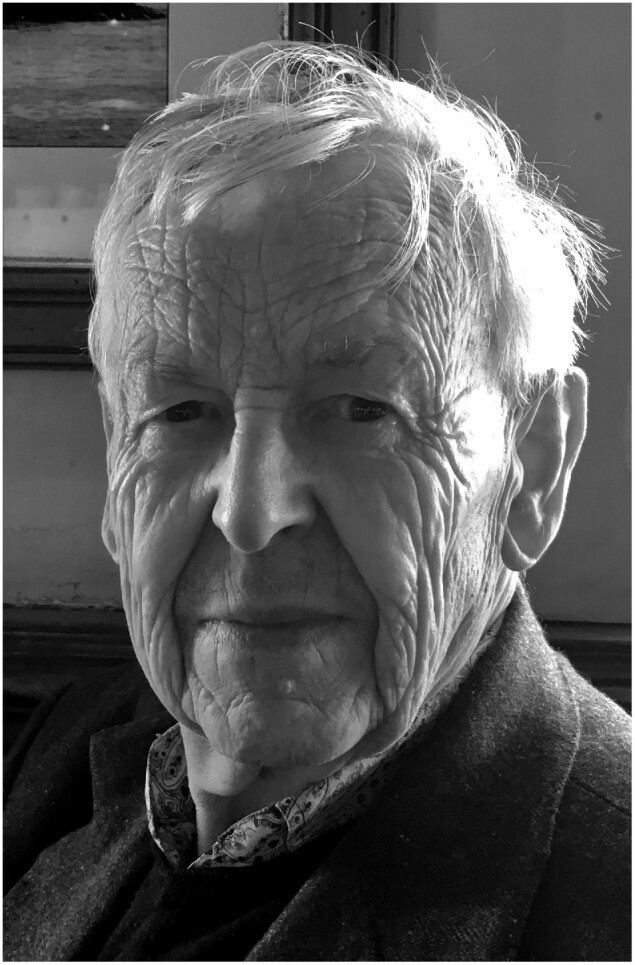
Photo of Colyn Crane-Robinson, taken by his son Max Crane-Robinson ca 2018.

Scientific colleagues and close friends were saddened to learn about the death of Colyn Crane-Robinson (Figure 1), who died on 5 March 2023. From a lifetime of enthusiastically and gregariously applied dedication, Colyn Crane-Robinson made substantial contributions to our understanding of nucleic acid structures and DNA–protein interactions. Numerous mechanistic insights were due to his ability to develop, adapt and improve existing methodologies, such as that of chromatin immunoprecipitation. Such innovative science resulted in many publications, including several landmark papers published in *Nucleic Acids Research* (1–13). Much of his work was pioneering and often highly collaborative, and it revealed unexpected truths by shedding new light on underlying principles.

Colyn Crane-Robinson was born in the North of England, in Sale, now part of Greater Manchester. He was greatly influenced by his father, a chemist, and received his secondary education at a school run by Quakers in Ackworth, West Yorkshire. Fortunately, science (physics, chemistry, biology) was at the core of the curriculum and Crane-Robinson was lucky to receive one-to-one tutorials from an engaging chemistry teacher, Mr G. Phillips Harris. Indeed, Phillips Harris introduced Colyn not only to the wonders of chemistry, but also of music, including compositions by Bartok, Stravinsky, Schönberg, Berg, Webern, and especially, Mahler. In his O-levels examinations, Crane-Robinson was successful, with one notable exception - English Language. This was a problem because a pass in the subject was required at the time for university entry. Luckily for Crane-Robinson, his English teacher took him in hand and presented him with a copy of ‘Plain Words’ by Sir Ernest Gowers, which was published in 1948 to stimulate civil servants to write simple and clear English. Crane-Robinson subsequently passed his English Language O-level and it seems reading the book left a life-long impression on the young man, as his scientific writing thereafter was always lucid, even when dealing with complex topics.

Crane-Robinson gained entry to the University of Oxford (Christ Church) in 1954, ultimately graduating with a First-Class Honours degree in Chemistry. Afterwards, he registered for a D.Phil degree, undertaking research in the laboratory of Harold Warris Thompson, a physical chemist and spectroscopist and an elected Fellow of the Royal Society (and who had also taught G. Phillips Harris, Crane-Robinson's chemistry teacher).

In the 1960s, the typical destination for newly qualified PhD students was the USA. Yet, Crane-Robinson decided to go eastwards, an idea perhaps engendered by his several visits to Yugoslavia and his efforts with the Serbo-Croatian language. As it happened, Thompson was at that time Foreign Secretary of the Royal Society and he knew that the UK government had signed a cultural treaty with the USSR in 1958 that provided for reciprocal transfer of scientists (for a stay of 1 year). In 1960, Crane-Robinson was successful in securing one of two more senior posts. His destination was the Institute for Higher Molecular Weight Compounds (IVS), a polymer chemistry institute, located in Leningrad, right in the centre of the city with line-of-sight view of the Winter Palace. The Institute's remit was not only the chemistry of polymer synthesis but also the physico-chemical aspects of key processes and their related theories. The head of the physico-chemical laboratory was Mikhail Volkenstein, and together with co-workers Oleg Ptitsyn and Tatiana Birshtein, they exerted a positive influence on Crane-Robinson, not least due to their interest in biopolymers, principally proteins. Crane-Robinson's work in the Volkenstein laboratory involved Raman spectroscopy of several polymers. During this time, Crane-Robinson ‘was catching the biopolymers bug’, as he described it. Following his stay in the USSR, Crane-Robinson initially joined Courtaulds (a UK polymer company), but soon afterwards its research division was closed forcing him to move on to Portsmouth Polytechnic (now University of Portsmouth). Crane-Robinson joined in 1963 as a Lecturer, later becoming a Professor, before Professor Emeritus upon his retirement in 2000.

At Portsmouth, much in collaboration with Morton Bradbury and Henry W. Rattle (one of Crane-Robinson's first PhD students), he initially used nuclear magnetic resonance to investigate the dynamic folding of peptides and proteins, such as helix-coil transitions, resulting in a string of high profile publications (14–18). Similar analyses were then successfully applied to histones, some of the most conserved cellular proteins, pivotal for packaging the nuclear genome (19–21). This work underpinned Crane-Robinson's future drive to better understand histone-DNA interactions, chromatin and the role of histone modifications and histone variants in gene regulation. Early highlights of this histone-DNA work include the structural analysis of histone H1-nucleosome interactions, especially on the location of histone H1 on the nucleosome (22–25). Another important study centred around characterisation of the H3/H4 nucleosomal kernel as a fundamental chromatin unit, published in *Nucleic Acids Research* (1). Crane-Robinson's group also studied another important class of chromatin proteins – those that contain HMG-boxes. This work led to the solution structure of a HMG-box (4) which revealed a conserved DNA-binding (and bending) motif found in various chromatin and transcription factors, such as the sex-determining factor, SRY. The work on HMG-box containing proteins also led to the analysis (with Peter Privalov) of the thermodynamics of the HMG-box protein interaction with DNA (8,26,27).

Although Crane-Robinson interacted with laboratories around the world, his most widely recognised scientific contribution was from a ‘home grown’ project, initiated and performed solely in ‘his’ Biophysics Unit at the University of Portsmouth. Here, together with his PhD student Tim Hebbes, he developed native chromatin immunoprecipitation, where antibodies recognizing modified histones are used to enrich specific regions of the genome. This technique, and its later derivatives, such as ChIP-seq (chromatin immunoprecipitation sequencing) have been invaluable in making functional connections between histone modifications and DNA processes, particularly gene expression (3,28–32). Chromatin immunoprecipitation has now become a mainstay for analysing epigenomes, helping us to understand the functional genome. In this way, it contributes to our understanding of cell type, cellular function and gene regulation mechanisms in health and disease. Indeed, Crane-Robinson recognized this contribution; in his own words, he claimed to be 'the principal midwife' at the birth of chromatin immunoprecipitation (33).

Chromatin immunoprecipitation remains an invaluable technique for the modern investigator. For instance, it is used to help identify chromatin features that characterise or define particular regulatory DNA elements, such as enhancers and promoters. Crane-Robinson contributed to this quite early on with two subsequent studies published in *Nucleic Acids Research*. The first paper highlights acetylation of histone variant H2A.Z as a hallmark of the promoter proximal region and 5′ end of active genes, while the unacetylated form is depleted from both active and inactive genes (10). The second paper showed that developmental activation of a paradigm gene (the *lysozyme* gene) in chicken macrophage cells is linked to core histone acetylation at its enhancer elements (11), connecting histone acetylation and enhancer function.

Crane-Robinson was not someone to rest on his laurels, and he continued to push forward right up to and including the last few weeks of his life. Indeed, it is a testament to his dedication that he continued to contribute very original and fundamental work in the decades following his formal retirement in 2000. Most of his latest papers, all in collaboration with Peter Privlov, examined the forces maintaining the DNA double helix and protein folding and the role of hydration and enthalpy versus entropy-driven processes in this context.

Privalov, an experimentalist, developed differential scanning calorimetry (DSC) to measure the thermodynamic principles of protein and nucleic acid folding (34). Crane-Robinson collaborated with Privalov from the early 1980s and he described their relationship as one of an apprentice (him) and master (Privalov). However, to most colleagues familiar with the situation, it was clear that their interaction was mutually beneficial. This synergy propelled the two of them to fruitfully explore the interactions between DNA and transcription factor DNA binding domains (DBDs).

With regards to DNA duplex thermodynamics, their findings challenged the generally held view that G-C base pairs stabilise duplexes more than A-T base pairs because they possess an additional Hydrogen-bond (three versus two). These findings are summarised in their last paper, published in 2022 (35), which built on work published over the last few years, (13,36,37). A key study in this string was the Vaitiekunas et al. paper (13), published in *Nucleic Acids Research*, where calorimetric experiments measuring the energetics of DNA duplex formation led to the provocative proposal that the intrinsic enthalpies of G-C and A–T base pairs are very similar. An A–T base pair gains this extra enthalpic stabilisation from one or two water molecules that are strongly fixed by its polar groups in the minor groove. So, where does the apparent overall stabilizing effect of G-C base pairs stem from? As with all enthalpy/entropy trade-offs, it must be in the entropy. They propose that on melting (breaking) an A–T base pair, the immobilised water is released, and it gains a large amount of favourable translational entropy in the process, accounting for the difference. It is astonishing to see how carefully planned and executed thermodynamic analysis of DNA melting can provide such fundamental insights nearly 70 years after the structure of DNA was solved.

Privalov, Crane-Robinson and collaborators also challenged the previously held belief that DNA energetics are temperature-independent. Using careful measurements with several short duplexes, they showed this is not the case, with the hydrating water once again playing a critical role (13,38). Interestingly, they also showed that similar conclusions can be drawn about the principles of alpha-helices folding in proteins (36).

Crane-Robinson never really stopped being scientifically active and he was in the lab until the very end. Indeed, we had stimulating discussions with him just a few weeks before his death. He was a fascinating, original personality, yet strikingly humble in the face of science. He was a great mentor and generous with insights and thoughts. He was witty with remarks, anecdotes and sometimes flamboyant in his dress style, often being the first and ‘best’ dressed person on the dance floor at conference parties.

He simply refused to retire and continued to walk through the wonderland of scientific discovery until the very end. Crane-Robinson not only gained pleasure from fruitful collaborations and intense discussions, but he considered them critical for scientific progress. He wrote: ‘Science is a conversation: its participants tell each other about their results, and the building rises from their combined efforts’ (34). We will miss this conversation with him.
